# Development of Multiple Autoimmune and Endocrine Disorders Following COVID-19 Vaccination: A Case Report

**DOI:** 10.7759/cureus.95744

**Published:** 2025-10-30

**Authors:** Abdullah M Almutairi, Mushabab A Alshahrani, Khalid A Albalawi, Abdullah S Basaba, Sabah A Roybaa

**Affiliations:** 1 Internal Medicine, Diabetes and Endocrinology Division, Security Forces Hospital, Riyadh, SAU; 2 Department of Internal Medicine, Prince Faisal bin Khalid Cardiac Center, Abha, SAU; 3 Endocrinology Department, King Salman Armed Forces Hospital, Tabuk, SAU; 4 Department of Internal Medicine, King Abdulaziz Hospital, Taif, SAU; 5 Department of Internal Medicine, Ahad Rafidah General Hospital, Ahad Rafidah, SAU

**Keywords:** astrazeneca, central adrenal insufficiency, covid-19 vaccine, hypothyroidism, lambert-eaton myasthenic syndrome, vitiligo

## Abstract

Coronavirus disease 2019 (COVID-19) vaccines have been instrumental in mitigating the pandemic, but rare post-vaccination immune-related events have been reported. Due to the brief trial period and the pressing need to begin vaccination, adverse events have been documented globally in real-time data format. There have been recent reports of new inflammatory diseases or flare-ups of immune-mediated diseases after vaccinations all over the world. Despite the low prevalence of vaccination-related side effects, it is still important to report and identify the endocrine diseases linked to the COVID-19 vaccine to facilitate prompt diagnosis and treatment. We present the case of a previously healthy 42-year-old man who developed multiple autoimmune and endocrine conditions following administration of the AstraZeneca COVID-19 vaccine. Over the course of three years following vaccination, he developed vitiligo, herpes zoster (HZ), hypothyroidism, Lambert-Eaton myasthenic syndrome (LEMS), and central adrenal insufficiency. The clinical course began with skin depigmentation and rash following the initial doses, progressing to neuromuscular and endocrine abnormalities. Comprehensive investigation and multidisciplinary treatment led to stabilization of his condition. This case highlights a potential temporal association between COVID-19 vaccination and the development of multiple autoimmune and endocrine disorders. Further investigation and long-term monitoring are necessary to better understand the mechanisms and outcomes in similar cases.

## Introduction

The global pandemic of coronavirus disease 2019 (COVID-19) has had a devastating impact on individuals and the economy globally [1]. The COVID-19 vaccine has been demonstrated in studies to effectively resist strains of the disease-causing virus, severe acute respiratory syndrome coronavirus 2 (SARS-CoV-2) [2]. Clinical trials have proven their effectiveness and safety. However, due to the short trial duration and the pressing need to begin vaccination, adverse events have been documented globally in real-time data format. There have been recent reports of immune-mediated disease flares or newly developed inflammatory diseases after vaccination administration all over the world [3].

Endocrine dysfunction is among the adverse effects reported in the literature after SARS-CoV-2 vaccination and mainly involves the thyroid gland, islets, pituitary gland, and adrenal gland. An "endocrine phenotype" of COVID-19 has progressively gained clinical concerns due to the endocrine system's involvement in the virus. These symptoms include pituitary apoplexy, thyroid dysfunction, hyperglycemia and diabetes, adrenal insufficiency, and hypogonadism. Similar to how COVID-19 infections impair endocrine organs, COVID-19 vaccinations also cause endocrine dysfunction [4]. Possible mechanisms include autoimmune activation by adjuvants, molecular mimicry between viral and endocrine antigens, and systemic inflammatory responses causing transient metabolic or hormonal disturbances. Antigenic similarities between the SARS-CoV-2 spike protein and human proteins can lead to anti-SARS-CoV-2 antibodies cross-reacting with human antigens, including extractable nuclear antigens, nuclear antigens, and myelin basic proteins. In cases of hyper-reactogenicity, vasodilators and cytokines enter the bloodstream, triggering a systemic inflammatory response syndrome [5].

This case report presents a man who experienced several autoimmune and endocrine diseases after receiving the AstraZeneca COVID-19 vaccine, illustrating the possibility of complicated, multi-system consequences after immunization.

## Case presentation

A 42-year-old man with no medical history was in his usual state of health when he received his first dose of the AstraZeneca COVID-19 vaccine on March 30, 2021. Two months later, on May 15, 2021, he began to experience new scattered skin depigmentation on his scalp, neck, and back, which subsequently spread across his entire body and was accompanied by hair discoloration. He was evaluated by a dermatologist, who diagnosed him with vitiligo and prescribed monobenzone cream (20%) to be used daily for six months. He received his second dose of the AstraZeneca COVID-19 vaccine on September 28, 2021, followed by a third dose of the Pfizer COVID-19 vaccine on February 13, 2022.

On August 13, 2022, the patient developed severe pain in the thoracic and lumbar regions, which was followed by a skin rash characterized by herpetiform vesicles. An infectious disease specialist diagnosed him with herpes zoster (HZ), and he was treated with acyclovir and a short course of prednisolone.

Two months later, on October 20, 2022, he presented with weakness, fatigue, cold intolerance, constipation, and dry skin. He sought medical attention at a family medicine clinic, where a thyroid function test was performed and results shown in Table 1, an adequate thyroid-stimulating hormone (TSH) of 5.28 uIU/mL, and low free T4 of 3.3 pmol/L, suggesting central hypothyroidism, and he was started on levothyroxine (25 mg daily), which was gradually increased to 100 mg daily.

**Table 1:** Summary of laboratory findings in a case of autoimmune disorders following COVID-19 vaccination

Test	Result	Reference range
Thyroid function tests		
- Thyroid-stimulating hormone (TSH)	5.28 uIU/mL	0.27-4.20 uIU/mL
- Free thyroxine (free T4)	3.3 pmol/L	12.0-22.0 pmol/L
Cortisol levels		
- Random cortisol	80 nmol/L	Normal: >138 nmol/L
- High-dose cosyntropin stimulation		
- Baseline cortisol	81 nmol/L	Normal response >500 nmol/L
- 30 minutes	231 nmol/L	
- 60 minutes	299 nmol/L	
Adrenocorticotropic hormone (ACTH)	2.82 pg/mL	7.20-63.30 pg/mL
Additional hormonal workup		
- Prolactin	2.03 uIU/mL	86.00-324.00 uIU/mL
- Follicle-stimulating hormone (FSH)	0.3 IU/L	Up to 12.4 IU/L
- Luteinizing hormone (LH)	0.30 IU/L	Up to 8.6 IU/L
- Testosterone	0.09 nmol/L	8.64-29 nmol/L

On November 8, 2022, the patient visited the emergency department, reporting progressive symptoms of unsteadiness and worsening upper and lower limb weakness, exacerbated by exercise but improved with rest, weight loss, loss of appetite, and a sensation of incomplete bowel evacuation. He was admitted under the care of a neurology team for further investigation. Nerve conduction studies revealed asymmetrical axonal neuropathy, predominantly affecting sensory nerves in both upper and lower limbs. A computed tomography (CT) scan and magnetic resonance imaging (MRI) of the brain were unremarkable, and the patient remained hemodynamically stable during his admission. He was discharged in stable condition with a plan for outpatient follow-up to complete the workup.

On January 21, 2023, during a follow-up with neurology, he continued to experience fatigue while eating and talking, especially at the end of the day, along with progressive upper and lower limb weakness and ptosis. Further investigations revealed negative acetylcholine receptor antibodies (ACh Ab) but positive voltage-gated calcium channel antibodies (VGCC Ab). He was diagnosed with Lambert-Eaton myasthenic syndrome (LEMS). Chest CT revealed no definite lung mass lesion. He began treatment with pyridostigmine (30 mg three times daily) and intravenous immunoglobulin (IVIG) every two months. The patient gradually improved and continued regular follow-ups with his neurologist.

On February 20, 2024, the patient visited a primary healthcare center, reporting dizziness and a near syncopal episode. He stated that he did not lose consciousness or experience abnormal movements. This episode lasted for one minute and was associated with nausea, vomiting, and abdominal pain. Upon examination, his blood pressure was found to be hypotensive, prompting his transfer to the emergency department, where he was resuscitated with intravenous fluids.

On presentation, his pulse was 116 beats per minute, his blood pressure was 91/55 mm Hg, and his weight was 75 kg. He exhibited decreased muscle strength, decreased lean body mass, and skin depigmentation consistent with vitiligo. There was absent axillary hair distribution, no hirsutism, no central obesity, and normal external genitalia with absent pubic hair; both testes were present in the scrotum but were of small volume (<15 mL). He appeared fatigued, although the remainder of the physical examination was unremarkable. Low cortisol and adrenocorticotropic hormone (ACTH) and an inadequate cosyntropin response suggest secondary adrenal insufficiency. The patient also reported symptoms of low libido and erectile dysfunction over the past two years, including a lack of morning erections, no sexual intercourse, and absence of ejaculation for two years with low LH, FSH, and testosterone, suggesting hypogonadotropic hypogonadism. He had not mentioned these symptoms to his doctors before, failing to recognize his condition. There was no history of central nervous system tumors or infections, mumps, prior radiation therapy, chemotherapy exposure, traumatic brain injury, testicular trauma, chronic intense exercise, use of testosterone or anabolic steroids, or sleep apnea. The patient did not report polyuria, nocturia, polydipsia, or laboratory findings suggestive of diabetes insipidus. All laboratory findings are shown in Table 1.

**Figure 1:**
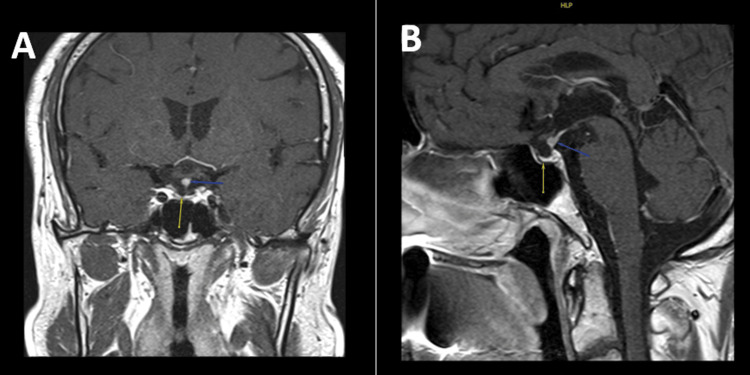
MRI pituitary protocol (A: coronal plane, B: sagittal plane) showed a focal, rounded area of widening seen involving the proximal part of the pituitary stalk, which showed homogeneous enhancement measuring around 4 mm.

The patient was treated with intravenous hydrocortisone and subsequently transitioned to prednisolone (5 mg in the morning and 2.5 mg in the evening). He was evaluated by a urologist and began treatment with human chorionic gonadotropin (HCG) (250 mcg subcutaneously twice a week) because he is concerned about fertility. A dedicated MRI of the pituitary revealed a focal, rounded area of widening seen involving the proximal part of the pituitary stalk; it showed homogeneous enhancement measuring around 4 mm. The rest of the pituitary stalk looks normal. The pituitary glandular tissue is thinned, but it showed normal enhancement and no evidence of a focal lesion and normal parasellar and suprasellar space. The rest is unremarkable. In summary, the MRI of the pituitary showed focal thickening seen in the pituitary stalk (Figure 1).

Table 2 shows the case timeline and summary.

**Table 2:** Case timeline and summary

Diagnosis	Time of onset	Main clinical manifestations	Diagnostic findings / tests	Treatment given
Vitiligo	May 2021	Scattered depigmented macules on the scalp, neck, and back spreading to the whole body; hair discoloration	Clinical diagnosis by a dermatologist	Monobenzone cream 20% daily for six months
Herpes zoster	August 2022	Severe thoracic and lumbar pain followed by herpetiform vesicular rash	Clinical diagnosis by an infectious disease specialist	Acyclovir and a short course of prednisolone
Central hypothyroidism	October 2022	Fatigue, weakness, cold intolerance, constipation, and dry skin	Low free T4 with inappropriately normal/elevated TSH	Levothyroxine (titrated to 100 µg daily)
Lambert-Eaton myasthenic syndrome (LEMS)	January 2023	Muscle weakness (upper and lower limbs), fatigability, ptosis, and unsteadiness	Nerve conduction study: asymmetrical axonal neuropathy; positive VGCC antibodies; Negative AChR antibodies	Pyridostigmine 30 mg TID and IVIG every two months
Secondary adrenal insufficiency and hypogonadotropic hypogonadism	February 2024	Dizziness, fatigue, hypotension, low libido, erectile dysfunction, and absent body hair	Low cortisol and ACTH, inadequate cosyntropin response; low LH, FSH, testosterone; pituitary MRI: stalk thickening	IV hydrocortisone → oral prednisolone; hCG 250 µg SC twice weekly

## Discussion

This case report describes the complex medical history of a 42-year-old man who had no prior medical disorders and experienced several health concerns after receiving the AstraZeneca COVID-19 vaccination. The sequence of events highlights the possibility of vaccine-related problems and raises questions regarding the pathophysiological mechanisms involved.

Different side effects of vaccinations were reported. A study conducted by Faksova et al. [6] reaffirmed the previously detected rare safety concerns, including myocarditis and pericarditis following messenger RNA (mRNA) vaccines (Pfizer and Moderna), as well as Guillain-Barré syndrome and cerebral venous sinus thrombosis (CVST) after viral vector vaccines (AstraZeneca). In the reported case, the patient's initial presentation of vitiligo two months after receiving his first dose of the AstraZeneca vaccine suggests a possible autoimmune reaction. Similarly, Kasmikha et al. [7] identified 17 patients with vitiligo, with 15 cases presenting new-onset or worsening vitiligo following COVID-19 vaccination. There is growing evidence that vaccines might cause autoimmune disorders in predisposed persons due to molecular mimicry, which occurs when vaccine components closely resemble human proteins, resulting in cross-reactivity and ultimately autoimmunity [8]. In this example, similarities between the SARS-CoV-2 spike protein and human proteins may have contributed to the development of vitiligo.

Following the administration of the second dose of the AstraZeneca vaccine, the patient experienced severe thoracic and lumbar pain along with a herpetiform vesicular rash, leading to a diagnosis of shingles. It is important to note that viral reactivations have also been reported following COVID-19 vaccination, including reactivation of varicella zoster virus (VZV). While the precise triggers for reactivation remain unclear, factors such as a weakened immune system, aging, or stress may contribute. Furthermore, HZ infection can cause complications such as postherpetic neuralgia, secondary bacterial infections, or ophthalmic issues. Bostan et al. reported a case that developed five days after the application of the inactivated COVID-19 vaccine; some stinging and painful pimple-like lesions had appeared involving the left mammary region. Dermatological examination showed some crusted, hemorrhagic vesicles upon an erythematous base occupying an area, and the final diagnosis was HZ [9].

In this case, the findings of negative acetylcholine receptor antibodies but positive voltage-gated calcium channel antibodies point toward LEMS, an autoimmune disorder that often co-occurs with malignancies but can also arise without an identifiable cause. Since the patient is less than 50 years old, a CT scan revealed no definite lung masses, and the patient is not a smoker, paraneoplastic LEMS was excluded. In the same vein, a systematic review conducted by Tugasworo et al. [10] found that COVID-19 infection can elevate the risk of new-onset myasthenia gravis (MG), trigger myasthenic crises, and lead to respiratory failure, all of which contribute to higher mortality rates in MG patients, particularly due to the cytokine storm associated with the infection. The structural similarity between the acetylcholine receptor and the SARS-CoV-2 receptor, activation of latent autoimmune diseases, and hyperinflammation (such as multisystem inflammatory syndrome in children) may provide a possible explanation for these effects. The patient developed overt hypothyroidism, presenting with fatigue, cold intolerance, and neurological symptoms like weakness and unsteadiness. Laboratory results suggested central hypothyroidism with elevated TSH and low free T4 levels. The diagnosis of secondary adrenal insufficiency was based on low adrenocorticotropic hormone (ACTH) levels and an inadequate cortisol response to the short Synacthen test. The reports of thyroid dysfunction, diabetes, and adrenal insufficiency following vaccination suggest a potential complex interaction between the immune response and endocrine health. A systematic review by Zarkesh et al. found that COVID-19 patients often have abnormal thyroid function test results compared to healthy controls, including decreased levels of TSH, T3, and T4, along with increased anti-thyroid antibodies [11]. The first case report of isolated adrenocorticotropic deficiency potentially associated with COVID-19 immunization was by Morita et al. [12].

Vaccines, particularly those for SARS-CoV-2, are designed to generate a strong immune response targeting specific viral epitopes. However, in some cases, this immune activation may lead to unintended effects on the endocrine system. These effects could manifest as disruptions in thyroid function, glucose regulation, and adrenal gland activity, indicating a need for further research to fully understand the mechanisms underlying these adverse events [13].

A hormonal evaluation revealed low testosterone, follicle-stimulating hormone (FSH), and luteinizing hormone (LH) levels, contributing to sexual dysfunction. Treatment involved hydrocortisone followed by prednisolone, with regular monitoring for treatment efficacy. FSH and HCG were initiated by urology services to address testosterone deficiency and improve sexual health and fertility. Indeed, research has demonstrated that COVID-19 can affect multiple endocrine organs, including the pituitary, thyroid, pancreas, adrenals, and gonads, due to the expression of the angiotensin-converting enzyme 2 (ACE2) receptor, which facilitates SARS-CoV-2 attachment and leads to cellular damage. The involvement of the endocrine system in COVID-19 has garnered increasing clinical attention, leading to the recognition of an "endocrine phenotype" of the disease [14,15]. This phenotype encompasses a range of conditions such as pituitary apoplexy, thyroid dysfunction, hyperglycemia and diabetes, adrenal insufficiency, and hypogonadism. Several endocrine problems were reported after COVID-19 vaccination, like type 1 diabetes mellitus, according to a systematic review of case reports by Alsuadais et al. [16]. Aliberti et al. reported a case of pituitary apoplexy, a rare but serious endocrine emergency characterized by the sudden hemorrhage or infarction of the pituitary gland [17]. Increasing the risk of thyroid eye disease case series by Muller et al. [18], COVID-19 vaccine-associated subacute and silent thyroiditis reported by Saha et al. [19], and thyrotoxicosis reported by Lee et al. [20].

## Conclusions

This case report shows the complex interplay between COVID-19 vaccination and the development of multiple autoimmune and endocrine disorders in a previously healthy adult. The patient experienced a range of conditions, including vitiligo, HZ, LEMS, hypothyroidism, and secondary adrenal insufficiency, after receiving the AstraZeneca COVID-19 vaccine. These findings raise critical issues about the pathophysiological processes of vaccine-induced autoimmunity, as well as the involvement of several systems. The patient's presentation emphasizes the significance of ongoing monitoring and research into the possible long-term effects of COVID-19 vaccinations, particularly concerning autoimmune and endocrine problems. Since it is only one case, more research is needed to better understand these relationships and inform future immunization tactics in susceptible populations.
